# Effects of Chest Physical Therapy in Patients with Non-Tuberculous Mycobacteria

**DOI:** 10.23937/2378-3516/1410065

**Published:** 2017-01-21

**Authors:** Ashwin Basavaraj, Leopoldo Segal, Jonathan Samuels, Jeremy Feintuch, Joshua Feintuch, Kevin Alter, Daniella Moffson, Adrienne Scott, Doreen Addrizzo-Harris, Mengling Liu, David Kamelhar

**Affiliations:** 1 Division of Pulmonary, Critical Care and Sleep Medicine, New York University School of Medicine, USA; 2 Division of Biostatistics, Department of Population Health and Environmental Medicine, New York University School of Medicine, USA

**Keywords:** Bronchiectasis, Non-tuberculous mycobacteria, Chest physical therapy, Airway clearance

## Abstract

Antibiotic therapy against non-tuberculous mycobacteria (NTM) is prolonged and can be associated with toxicity. We sought to evaluate whether chest physical therapy (PT) was associated with clinical improvement in patients with NTM not receiving anti-mycobacterial pharmacotherapy.

A retrospective review of 77 subjects that were followed from June 2006 to September 2014 was performed. Baseline time point was defined as the first positive sputum culture for NTM; symptoms, pulmonary function, and radiology reports were studied. Subjects were followed for up to 24 months and results analyzed at specified time points.

Half of the subjects received chest PT at baseline. Cough improved at 12 (p = 0.001) and 24 months (p = 0.003) in the overall cohort when compared with baseline, despite lack of NTM antibiotic treatment. Cough decreased at 6 (p = 0.01), 9 (p = 0.02), 12 (p = 0.02) and 24 months (p = 0.002) in subjects that received chest PT. Sputum production also improved at 24 months in the overall cohort (p = 0.01). There was an increase in the percent change of total lung capacity in subjects that received chest PT (p = 0.005).

Select patients with NTM may have clinical improvement with chest PT, without being subjected to prolonged antibiotic therapy. Future studies are warranted to prospectively evaluate outcomes in the setting of non-pharmacologic treatment and aid with the decision of antibiotic initiation.

## Introduction

Although non-tuberculous mycobacteria (NTM) infection is not a reportable disease in the United States, recent data suggest that the prevalence of the disease is increasing in the United States [1,2], mainly due to increased clinical suspicion, improving diagnostic capability, and the aging of the general population [3]. Despite its increased recognition, however, a number of fundamental questions remain unanswered. One area of uncertainty is the ideal management of NTM patients. The 2007 American Thoracic Society/Infectious Disease Society of America statement provides general guidelines on the diagnosis and management of NTM [4]. However, except for cases with advanced and progressive disease, the statement does not provide clear recommendations about when treatment of NTM should be initiated, stating that the decision to start antibiotics should be based on potential risks and benefits of therapy for individual patients [4]. Antibiotic treatment is prolonged (at least 12 months of multiple antibiotics) and can be associated with significant toxicity, including auditory, visual and renal disease [5]. Delaying antibiotic treatment may be appropriate for some patients with stable or slowly progressive disease that may be difficult to treat [6]. Although large studies are lacking, non-pharmacological treatments have been utilized in the management of non-cystic fibrosis bronchiectasis [7,8]. Chest physical therapy (chest PT) includes manual chest PT, high-frequency chest wall oscillation devices (e.g. Vest^®^) and oscillating devices (e.g. Flutter^®^) that mobilize mucus. Recent data shows that oscillatory devices improve symptoms, exercise capacity [9], quality of life [10], pulmonary function and inflammatory markers in the non-cystic fibrosis bronchiectasis population [11]. However, data on the utility of non-pharmacological treatments in NTM pulmonary disease is sparse. We therefore performed a retrospective review of consecutive subjects with NTM seen during a period of 8 years who did not receive NTM antibiotic therapy, to evaluate whether chest PT was associated with a change in the natural history of their disease.

## Materials and Methods

### Study design

We performed a retrospective review on 77 subjects with culture confirmed NTM who did not receive NTM antimicrobial therapy during the study period (June 2006 to September 2014). Data collected included demographics, past medical history, clinical symptoms, pulmonary function, radiographic findings, sputum culture data, results from diagnostic testing for reflux, and therapy utilized. Inclusion criteria were subjects 21 years and older who had at least one positive Acid-Fast Bacilli (AFB) culture for NTM. Non-inclusion criteria were patients without culture confirmed NTM, pregnant patients, and those who were placed on antibiotic therapy for NTM at any time point during the study period. The study was approved by the Institutional Review Board at New York University School of Medicine (#S12-01987).

Baseline time point was defined as the first positive culture for NTM. Follow up time points included 3, 6, 9, 12, 18 and 24 months. During that time, standard clinical care was coordinated by one investigator (D.K.). The decision to hold antibiotic therapy (ie. stable or slowly progressive clinical status) and initiate conservative management with chest PT (ie. Flutter^®^, Acapella^®^, Vest^®^) was directed as per discretion of the treating physician. Routine pulmonary function assessment included spirometry, plethysmography, and diffusing capacity (Vmax; Sensor Medics; Yorba Linda, CA) according to standard American Thoracic Society/European Respiratory Society guidelines [12]. Data was compared with published normative values [13–15]. Images obtained included chest computerized tomography (CT), and interpretation was performed by board certified radiology as part of standard clinical care. AFB cultures were performed by the microbiology laboratory at New York University Langone Medical Center.

### Statistical analysis

Demographic and clinical data are expressed as Mean ± Standard Deviation or Median (Inter Quantile Range, IQR) for continuous variables and Count (Proportion, %) for categorical variables. McNemar’s test was utilized to evaluate changes in categorical variables over time. Comparisons of longitudinal continuous data amongst groups were made using the Kruskal-Wallis nonparametric one-way analysis of variance test, with subsequent post-hoc Mann-Whitney U testing between pairs. p-values < 0.05 were considered statistically significant. A linear mixed-effects model was performed to analyze the change of longitudinal pulmonary function over time assuming a random slope to account for within-subject correlation among repeated measurements and across-subject heterogeneity. Analyses were done using SPSS^®^ Statistics version 20.0 (IBM^®^ Corp, Armonk, NY) and R 3.2.2 [16].

## Results

### Baseline characteristics of the cohort

Table 1 describes the demographic and clinical characteristics of the subjects at baseline. Cough was the most common symptom (49%) at baseline, followed by sputum (39%). Although only 25% of subjects had heartburn symptoms at baseline, 52% had a diagnostic study (esophagram, laryngoscopies, barium swallow, or upper endoscopies) that suggested the presence of gastroesophageal reflux disease (GERD). Thus, there was a high prevalence of reflux (61%) in this cohort. Fifty-five subjects (71%) had more than one positive sputum culture for NTM. Half of the cohort received chest PT at baseline (47% with Acapella^®^ or Flutter^®^, 5% with Vest^®^, 1% with both modalities). Table 1 also shows that there were no major differences in demographics, past medical history, radiology, or intervention done between those that did and did not receive chest PT. However, chest PT was indicated more often in subjects that had symptoms of cough (p = 0.002), sputum (p < 0.001), heartburn (p = 0.02) or postnasal drip (p = 0.04).

### Longitudinal follow up of symptoms

**Figure 1:**
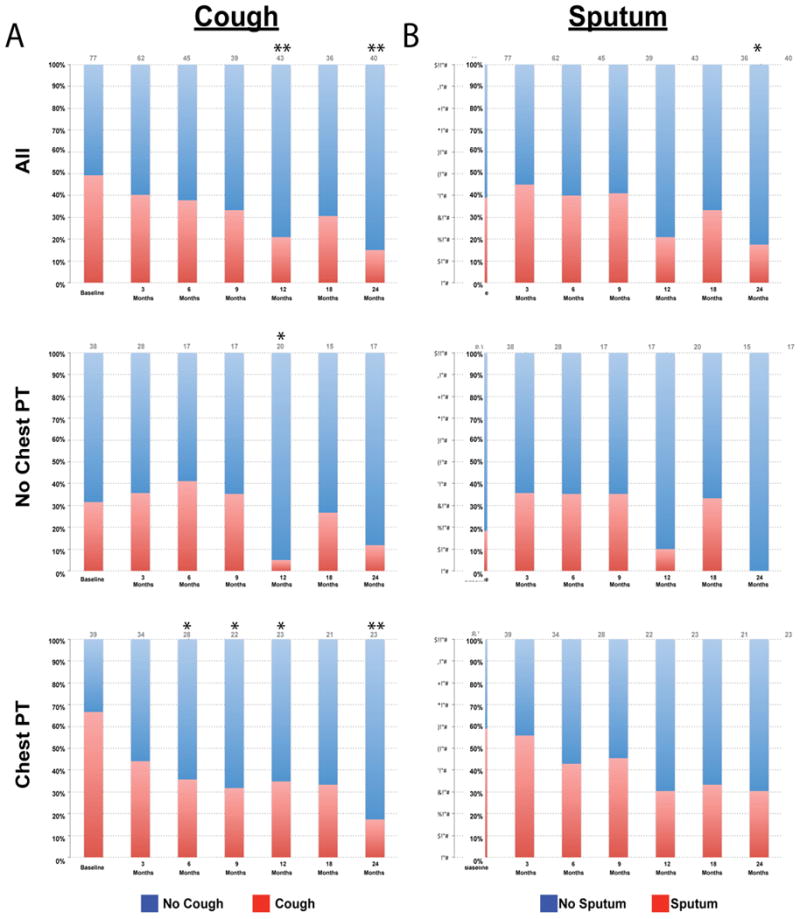
Change in cough and sputum over time A) Prevalence of cough at each time point for the overall cohort, chest physical therapy and no chest physical therapy groups. B) Prevalence of sputum at each time point for the overall cohort, chest physical therapy and no chest physical therapy groups. p value based on McNemar’s test. *denotes p < 0.05, **denotes p < 0.01.

Subjects were followed for up to 24 months. Cough significantly decreased at 12 (p = 0.001) and 24 months (p = 0.003) compared with baseline in the overall cohort, despite lack of NTM antibiotic treatment (Figure 1). In those who underwent chest PT, there was a significant decrease in cough at 6 (p = 0.01), 9 (p = 0.02), 12 (p = 0.02) and 24 months (p = 0.002) compared with baseline. There was a small but significant decrease in cough in subjects that did not receive chest PT at 12 months only (p = 0.03). Sputum production also improved significantly at 24 months compared with baseline in the overall cohort (p = 0.01). There was a trend towards reduced sputum production in the chest therapy group at 24 months; however this finding did not reach significance (p = 0.07). There was no significant change in sputum in subjects that did not receive chest PT. Other symptoms studied that had lower prevalence, including heartburn, postnasal drip, chest congestion, fatigue, hemoptysis, hoarseness, throat clearing and wheezing, did not show significant change over time in either group (data not shown).

In a subgroup analysis, we evaluated the association of cough and heartburn, and the influence of acid suppressive treatment on cough. Twenty-five percent of subjects had heartburn at baseline. The prevalence of heartburn among the subjects with cough was 74%, while the prevalence of heartburn in those that did not have cough was 26%. Chi- square analysis showed that at baseline, heartburn was associated with cough (p = 0.015). However, this association was lost at follow-up, possibly due to therapeutic interventions or dropout (data not shown). We therefore examined whether treatment of heartburn affected the prevalence of cough. In subjects that received heartburn treatment, there was a significant decrease in cough at 24 months (p < 0.001). However, there was also a significant decrease in cough at 12 months in subjects that did not receive heartburn treatment (p = 0.008). This suggests that some other intervention, possibly chest PT, was the reason for the decrease in cough.

### Longitudinal follow up of imaging

**Figure 2:**
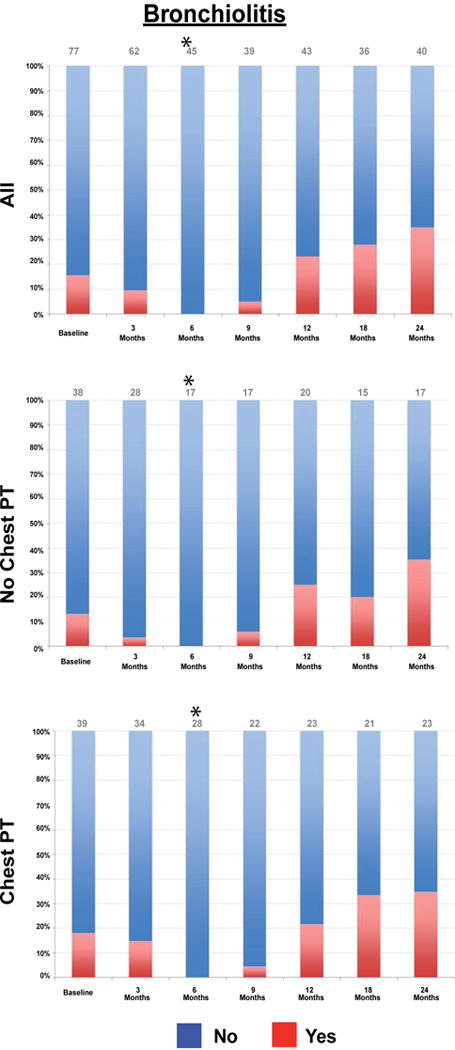
Change in bronchiolitis over time Prevalence of bronchiolitis at each time point for the overall cohort, chest physical therapy and no chest physical therapy groups. p value based on McNemar’s test. *denotes p < 0.05.

We then utilized CT reports from serial CT chest scans obtained in this cohort to evaluate the evolution of abnormalities observed. In the overall cohort, there was a significant decrease in bronchiolitis at 6 month follow-up (Figure 2, p < 0.001), bronchial wall thickening at 3 (p = 0.008) and 6 months (p < 0.001), mucoid impaction at 6 months (p = 0.03), ground glass opacities at 6 (p < 0.001) and 9 months (p = 0.01), nodules reported at 6 (p < 0.001), 9 (p < 0.001), 12 (p = 0.02) and 18 months (p = 0.03), and a significant decrease in hyperinflation at 6 months (p < 0.001).

In subjects that did not receive chest PT, there was a significant decrease in bronchiolitis at 6 months (p < 0.001), mucoid impaction at 6 months (p < 0.001), bronchial wall thickening at 3 (p = 0.016) and 6 months (p < 0.001) ground glass opacities at 6 (p < 0.001) and 9 months (p < 0.001), nodules at 6 (p < 0.001) and 9 months (p < 0.001) and hyperinflation at 6 months (p < 0.001).

In subjects that received chest PT, there was a significant decrease in bronchiolitis at 6 months (p < 0.001), bronchial wall thickening at 6 months (p = 0.016), ground glass opacities at 6 months (p = 0.031), nodules at 6 (p < 0.001) and 9 months (p < 0.001), and a decrease in hyperinflation at 6 (p < 0.001) and 9 months (p < 0.001). There was no change in mucoid impaction in those that received chest PT.

### Longitudinal follow up of pulmonary function

**Figure 3:**
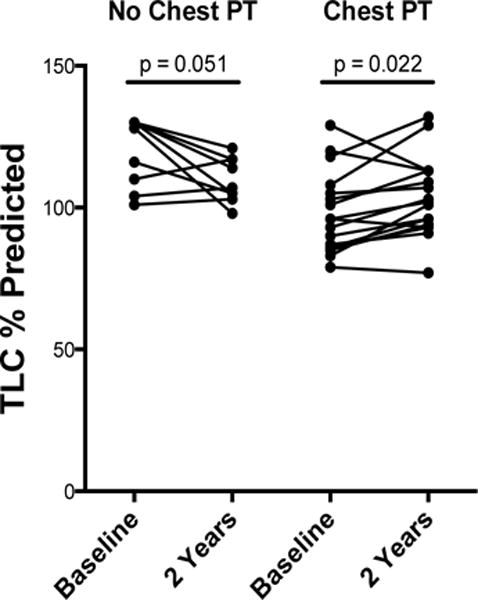
Change in TLC % predicted overtime Graph On left. Change in TLC% predicted at baseline and two year follow up in patients not receiving chest physical therapy. Graph On right. Change in TLC% predicted at baseline and two year follow up in patients receiving chest physical therapy. p value based on paired analysis.

Pulmonary function parameters were analyzed using paired analysis at the specified time points (Table 2). Chest PT was associated with an increase in FVC% predicted at 6 (4.1[−1.1, 16.9]% increase, p = 0.015) and 24 months (11.0[−1.5, 19.5]% increase, p = 0.028) compared with baseline. In contrast, minimal change was observed in subjects that did not receive chest PT. Similarly, other physiological parameters (FEV_1_/FVC%, TLC% predicted and RV% predicted) demonstrated significant changes over time in the chest PT group, while minimal changes occurred in subjects that did not receive chest PT (Figure 3).

To further evaluate lung function progression, we performed a linear mixed-effects model that accounts for changes over time in the chest PT and no chest PT groups, with percent change of pulmonary function parameters used as the dependent variable (Table 3). Importantly, there was a significant interaction between time and chest PT on the slopes of percent change in TLC (slope for no chest PT group −0.277, slope for chest PT group +0.293, p = 0.007 for the difference in slopes). After adjusting for the effect of heartburn on TLC % change, chest PT still showed a significant effect in change in TLC (the difference in slopes was 0.595, p = 0.005). Heartburn did have a mild independent effect on decreasing TLC % change (slope −0.443, p = 0.043). There was no significant change in either the chest PT and no chest PT groups for the percent change of other pulmonary function parameters studied in this analysis (data not shown).

## Discussion

The management of patients with NTM may present a challenge to clinicians. Patients may be subjected to prolonged antibiotic therapy [4], which has the potential for serious toxicity [5]. Alternatively, patients may be managed with close clinical monitoring and institution of non-pharmacologic therapy. Our data suggest that patients with NTM who have been placed on chest PT may have an improvement in cough, without being subjected to NTM antibiotic therapy. Our subgroup analysis suggests that in patients with symptomatic reflux, cough decreased over time irrespective of acid-suppressive therapy. This suggests that other interventions, including chest PT, may be the reason for improvement in cough. Based on radiology reports, there was an initial decrease in many of the radiographic findings reported in this study, irrespective of chest PT. However, this improvement was not sustained over 24 months. There was significant improvement in TLC% predicted in patients that received chest PT, even after adjusting for the presence of heartburn. Residual volume significantly worsened in subjects irrespective of chest PT, suggesting the possibility of progressive air-trapping in this cohort [17].

Airway clearance facilitated by chest PT allows for improvement in sputum expectoration, selected measures of lung function, and health-related quality of life [8]. The vicious cycle hypothesis states that bronchiectasis leads to increased mucus in the airways [18] with subsequent colonization of bacteria (including NTM) and resulting inflammation and airway damage. Clearance of this mucus may help to break this vicious cycle [19]. Clearance may help loosen mucus in the large airways and explain the improvement in symptoms and pulmonary function. However, given the progressive increase in air-trapping on pulmonary function seen in our study, it is possible that chest therapy may be less effective in clearance of mucus in the small airways. Our data also highlight the significant association of GERD and asymptomatic reflux with the NTM population as previously reported in other studies [20], seen in about 61% of our cohort. Previous studies have suggested the possible symptomatic benefit of anti-reflux treatment in bronchiectasis [21]. Our study did show a significant association with heartburn and cough, however with improvement in cough irrespective of acid-suppressive therapy. Heartburn also independently had a mild effect on reducing total lung capacity. This association with reflux and adverse consequences on pulmonary parenchyma has been described with other disorders including pulmonary fibrosis [22]. Further studies are needed to study this association of reflux and pulmonary disorders. Particularly, insight is needed on reflux as a potential predisposing factor to the development of bronchiectasis and NTM, and the effects of managing reflux in this population.

Limitations of this study include our retrospective design, small sample size and single-centered study. The 2007 American Thoracic Society guidelines suggest that two AFB sputum samples are needed for a diagnosis of NTM due to concern that a single positive induced sputum could represent a contaminant. Although one culture, patients may still present with radiographic signs and symptoms suggestive of NTM. In this cohort, 71% of the subjects had two or more positive cultures for NTM, and sub-analysis of this cohort showed similar trends. A larger prospective study will be needed to validate the findings described here. CT imaging results were also based on radiology reports and may not fully represent full extent of disease, as readings may have been limited by lack of clinical information. 14% of subjects were either on inhaled corticosteroids or bronchodilators, which may contribute to bias in the change in cough. While we did not find demographic differences between the chest PT and no chest PT groups, it is likely that a selection bias might be influencing our observations. Our focus on clinical outcomes in NTM managed without antimicrobial therapy prevents us from evaluating whether any beneficial effect of chest PT could be of additive benefit once anti-NTM treatment is initiated.

## Conclusion

In patients with *Mycobacterium Avium Complex* positive respiratory cultures in whom the physician has decided against initiation of anti-mycobacterial treatment, chest therapy may provide beneficial effects in respiratory symptoms and pulmonary function. Future prospective longitudinal studies are warranted to prospectively evaluate the natural progression of NTM disease and study clinical and treatment outcomes.

## Figures and Tables

**Table 1:** Baseline characteristics of cohort.

	Cohort	No chest PT	Chest PT	p value
	n = 77	n = 38	n = 39	
**Demographics**				
Age	67 ± 14	66 ± 16	68 ± 11	0.93
Body mass index	25 ± 5	24 ± 5	25 ± 5	0.65
Caucasian	69 (90)	35 (92)	34 (87)	0.47
Female	54 (70)	24 (63)	30 (77)	0.19
Smoking history	29 (38)	14 (37)	15 (38)	0.81
Pack years	6 ± 15	7 ± 15	5 ± 17	0.37
**Past medical history**				
Allergic rhinitis	11 (14)	7 (18)	4 (10)	0.31
Asthma	16 (21)	6 (16)	10 (26)	0.29
COPD	23 (30)	11 (29)	12 (31)	0.86
Hiatal hernia	15 (20)	6 (16)	9 (23)	0.42
Hypertension	33 (42)	17 (45)	16 (41)	0.74
Pneumonia	32 (42)	13 (34)	19 (49)	0.2
Reflux	47 (61)	23 (61)	24 (62)	0.93
Sinusitis	18 (23)	10 (26)	8 (21)	0.55
Tuberculosis	4 (5)	1 (3)	3 (8)	0.32
**Symptoms**				
Chest congestion	3 (4)	0 (0)	3 (8)	0.08
Cough	38 (49)	12 (32)	26 (67)	*0.002*
Fatigue	3 (4)	0 (0)	3 (8)	0.08
Heartburn	19 (25)	5 (13)	14 (36)	*0.02*
Hemoptysis	4 (5)	1 (3)	3 (8)	0.32
Postnasal drip	4 (5)	0 (0)	4 (10)	*0.04*
Sputum	30 (39)	7 (18)	23 (59)	< 0.001
Wheezing	4 (5)	1 (3)	3 (8)	0.32
**Mycobacterial culture**				
Myocobacterium avium complex	71 (92)	34 (89)	37 (95)	0.38
Mycobacterium gordonae	4 (5)	3 (8)	1 (3)	0.29
Mycobacterium kansasii	1 (1)	1 (3)	0 (0)	0.31
Mycobacterium xenopi	1 (1)	0 (0)	1 (3)	0.32
**CT chest findings**				
Bronchiectasis	22 (29)	12 (32)	10 (26)	0.56
Bronchiolitis	12 (16)	5 (13)	7 (18)	0.56
Bronchial wall thickening	19 (25)	8 (21)	11 (28)	0.47
Ground glass opacities	9 (12)	6 (16)	3 (8)	0.09
Mucoid impaction	11 (14)	3 (8)	8 (21)	0.11
**Intervention**				
Bronchodilator therapy	11 (14)	3 (8)	8 (21)	0.11
Inhaled corticosteroid	11 (14)	3 (8)	8 (21)	0.11
Proton pump inhibitor or H2 blocker	20 (26)	7 (18)	13 (33)	0.14

Data expressed as mean ± SD or N (%); p value based on chi-square for categorical variables and Mann-Whitney for continuous variables; COPD = chronic obstructive pulmonary disease; CT = computerized tomography.

**Table 2:** Percent change in pulmonary function over time in the overall cohort, chest PT and no chest PT groups.

		Baseline	3 months	6 months	9 months	12 months	18 months	24 months
		n = 77	n = 35	n = 30	n = 32	n = 27	n = 28	n=26
**FVC % predicted**	All	91 [76,106]	2.5 [−6.9,6.6]	4.0 [−1.3,17.4]**	4.7 [−5.7,15.6]	2.4 [0.0,10.0]*	9.2 [−8.1,18.4]	9.6 [0.5,19.5]**
	No Chest PT	100 [84,107]	−0.5 [−7.7,8.7]	3.9 [−1.9,28.0]	4.7 [−5.0,23.8]	2.4 [0.9,9.6]*	7.0 [−8.9,14.4]	8.3 [0.9,23.5]
	Chest PT	84 [69,103]	4.5 [−6.6,6.2]	4.1 [−1.1,16.9]*	4.0 [−6.4,19.7]	1.9 [−1.6,12.3]	11.8 [−8.9,21.1]	11.0 [−1.5,19.5]*
**FEV_1_ % predicted**	All	84 [67,94]	−10.7 [−22.8,0.0]	−5.9 [−15.9,0.3]	−3.8 [−14.5,6.7]	−9.6 [−24.4,1.0]	−7.5 [−17.4,3.0]	−6.4 [−15.6,4.4]
	No Chest PT	88 [72,96]	−11.6 [−23.8,−5.5]	−10.6 [−19.2,−1.2]	−13.7 [−21.4,10.7]	−9.8 [−25.8,1.0]	−7.5 [−21.3,1.8]	−7.0 [−12.4,−0.4]
	Chest PT	80 [65,92]	−10.6 [−22.5,5.9]	−5.8 [−15.6,1.1]	−7.8 [−14.2,6.7]	−6.9 [−23.6,1.6]	−5.5 [−16.8,6.6]	−3.5 [−16.3,8.0]
**FEV_1_/FVC %**	All	71 [64,77]	−25.0 [−36.4,0.0]	−21.1 [−36.0,−6.3]	−26.0 [−37.7,−6.2]	−24.3 [−30.8,−11.6]	−25.8 [−33.8,−10.8]**	−23.5 [−35.6,−5.9]*
	No Chest PT	71 [64,80]	−30.8 [−37.0,21.6]	−34.2 [−36.6,−22.4]	−33.3 [−38.4,−19.8]	−26.0 [−30.5,−3.5]	−25.0 [−40.5,−12.8]	−38.8 [−41.5,−18.7]
	Chest PT	71 [65,76]	−18.1 [−36.2,4.9]	−20.2 [−34.9,−2.5]	−23.3 [−36.7,−2.9]*	−21.2 [−40.0,−11.6]	−26.0 [−33.3,−9.6]**	−16.0 [−34.5,−2.3]
**TLC % predicted**	All	103 [93,115]	12.0 [0.8,31.8]	8.9 [−2.3,41.2]	19.5 [4.8,41.7]	20.9 [−1.0,40.6]	21.8 [1.5,44.4]	24.0 [−0.3,39.0]
	No Chest PT	112 [101,129]	8.2 [−3.1,24.1]	6.7 [2.9,62.0]	19.5 [−0.2,64.2]	23.2 [1.0,96.2]	13.4 [−4.8,−74.0]	13.6 [−2.9,63.2]
	Chest PT	100 [88,109]	22.7 [4.9,40.3]	11.1 [−2.7,39.5]	21.0 [5.7,38.2]	15.4 [−1.6,37.5]	24.3 [9.4,43.8]*	26.5 [8.3,38.7]*
**RV % predicted**	All	116 [99,150]	24.0 [−7.4,64.0]	21.8 [−1.4,86.3]	49.1 [10.3,68.3]	41.6 [−2.3,87.2]	32.5 [7.1,88.0]	46.3 [−3.2,77.5]
	No Chest PT	120 [109,163]	22.2 [−6.3,58.6]	4.8 [−5.6,97.3]	27.7 [−6.7,111.9]	64.6 [4.9,145.8]	12.5 [5.6,147.8]	28.2 [−9.9,104.0]*
	Chest PT	115 [92,131]	26.0 [−11.0,85.1]	31.1 [−26.6,82.6]	53.1 [24.3,68.3]*	31.4 [−3.8,72.6]	38.8 [14.7,87.3]	54.5 [10.8,73.6]

* p < 0.05;

** p < 0.01. p value based on Wilcoxon Sum Rank test for paired comparisons. Baseline data displayed as raw data. Follow up data displayed as percent change compared with baseline.

FVC = forced vital capacity; FEV_1_ = forced expiratory volume in one second; TLC = total lung capacity; RV = residual volume; PT = physical therapy.

**Table 3:** Estimates of the slope in percent change of lung function parameters from the linear mixed-effects model.

	No chest PT	Chest PT		
			Delta slope	p value
**Outcome**				
FVC % predicted	0.3009	0.495	0.1941	0.59
FEV_1_ % predicted	0.1541	0.17036	0.01626	0.96
FEV_1_/FVC %	−0.08721	−0.34021	−0.253	0.31
TLC % predicted	−0.2773	0.2937	0.571	0.007
RV % predicted	−0.1221	0.5781	0.7002	0.29

For each lung function parameter, the percent of change from baseline was used as dependent variable in the linear mixed-effects model. FVC = forced vital capacity; FEV_1_ = forced expiratory volume in one second; TLC = total lung capacity; RV = residual volume; PT = physical therapy.

## Abbreviations

AFB: Acid-fast bacilli
FEV1: Forced expiratory volume in one second
FVC: Forced vital capacity
GERD: Gastroesophageal reflux disease
NTM: Non-tuberculous mycobacteria
PT: Physical therapy
RV: Residual volume
TLC: Total lung capacity
